# Oleanolic acid attenuates TGF-β1-induced epithelial-mesenchymal transition in NRK-52E cells

**DOI:** 10.1186/s12906-018-2265-y

**Published:** 2018-07-04

**Authors:** Wei-ming He, Jia-qi Yin, Xu-dong Cheng, Xun Lu, Li Ni, Yi Xi, Gui-dong Yin, Guo-yuan Lu, Wei Sun, Ming-gang Wei

**Affiliations:** 1 Affiliated Hospital of Nanjing University of Chinese Medicine, Nanjing, 210029 Jiangsu China; 2 The First Affiliated Hospital of Soochow University, Suzhou, 215006 Jiangsu China; 3 Suzhou Hospital of Traditional Chinese Medicine, Suzhou, 215006 Jiangsu China; 4 Suzhou Municipal Hospital, Affiliated Hospital of Nanjing Medical University, Suzhou, 215006 Jiangsu China

**Keywords:** Oleanolic acid, EMT, TGF-β1, Nrf2, Klotho

## Abstract

**Background:**

Epithelial-to-mesenchymal transition (EMT) plays an important role in the progression of renal interstitial fibrosis, which finally leads to renal failure. Oleanolic acid (OA), an activator of NF-E2-related factor 2 (Nrf2), is reported to attenuate renal fibrosis in mice with unilateral ureteral obstruction. However, the role of OA in the regulation of EMT and the underlying mechanisms remain to be investigated. This study aimed to evaluate the effects of OA on EMT of renal proximal tubular epithelial cell line (NRK-52E) induced by TGF-β1, and to elucidate its underlying mechanism.

**Methods:**

Cells were incubated with TGF-β1 in the presence or absence of OA. The epithelial marker E-cadherin, the mesenchymal markers, α-smooth muscle actin (α-SMA), fibronectin, Nrf2, klotho, the signal transducer (p-Smad2/3), EMT initiator (Snail), and ILK were assayed by western blotting.

**Results:**

Our results showed that the NRK-52E cells incubated with TGF-β1 induced EMT with transition to the spindle-like morphology, down-regulated the expression of E-cadherin but up-regulated the expression of α-SMA and fibronectin. However, the treatment with OA reversed all EMT markers in a dose-dependent manner. OA also restored the expression of Nrf2 and klotho, decreased the phosphorylation of Smad2/3, ILK, and Snail in cells which was initiated by TGF-β1.

**Conclusion:**

OA can attenuate TGF-β1 mediate EMT in renal tubular epithelial cells and may be a promising therapeutic agent in the treatment of renal fibrosis.

## Background

The epithelial-mesenchymal transition (EMT) is a highly conserved process in which polarized, immobile epithelial cells are converted into motile mesenchymal cells with motile mesenchymal phenotypes [1–3]. The EMT is involved in various pathological processes, such as inflammation, fibrosis and tumorigenesis. Accumulating evidences show that EMT occurred in kidney plays an important role in the progression of renal interstitial fibrosis [4–7]. It is important to understand the mechanism to reverse EMT which is valuable establishing therapeutic strategies preventing progressive renal failure. Among many fibrogenic factors regulating renal fibrotic process, transforming growth factor-β1 (TGF-β1) is the key mediators that play critical roles in inducing EMT and renal fibrosis through the TGF-β1/Smads signal pathway [8–11]. The EMT process is characterized by the loss of epithelial markers such as E-cadherin and acquiring mesenchymal features, including α-smooth muscle actin (α-SMA) and fibronectin concomitantly with the increase in the expression of Snail protein [12–14], which is a zinc finger protein and functions as a core EMT transcription factors that plays critical roles in fibrosis via the down regulation of E-cadherin expression [15–17]. ILK plays critical role in renal tubular EMT process mainly by upregulating the expression of Snail [18–21]. In concert, the inhibition of TGF-β1 signaling has been included in several therapeutic approaches for preventing renal fibrosis [22, 23].

OA is a natural triterpenoid compounds that exist widely in food, medicinal herbs and other plants, which has recently attracted considerable attention for its antioxidant properties through the induction of Nrf2 activation [24–28]. Recently studies have also shown that OA is effective in protecting chemically induced liver injury in laboratory animals. Nrf2 is a basic leucine-zipper (bZip) transcription factor that protects cells and tissues from oxidative and electrophilic stress by activating antioxidant and detoxifying enzymes [29–31]. Recently study also shows that Nrf2 can restore the klotho expression and protect against renal fibrosis [32]. Klotho gene, a new anti-aging gene, is predominantly expressed in renal tubular epithelial cells [33]. Previous studies have found that the reduction of renal klotho gene expression is associated with the emergence and development of the pathological process of renal diseases [34]. The klotho protein can directly bind to the type-II TGF-β1 receptor and inhibit TGF-β1 binding to cell surface receptors, thereby inhibiting TGF-β1 signaling and reduced EMT responses [35]. We previously reported that the JiaWeiDangGui decoction, OA is one of the most effective components. OA reduces the accumulation of ECM in the kidneys of rats with Adriamycin-induced nephropathy [36]. However, protective effects of OA, an Nrf2 activator, against renal fibrosis induced by TGF-β1 has not been investigated.

In this study, we investigated the effects of OA on EMT of NRK-52E induced by TGF-β1 in vitro. We found that OA inhibited TGF-β1-induced EMT via upregulation of Nrf2 and klotho, it also inhibits the TGF-β1/Smads pathway.

## Methods

### Drug and reagents

**Fig. 1:**
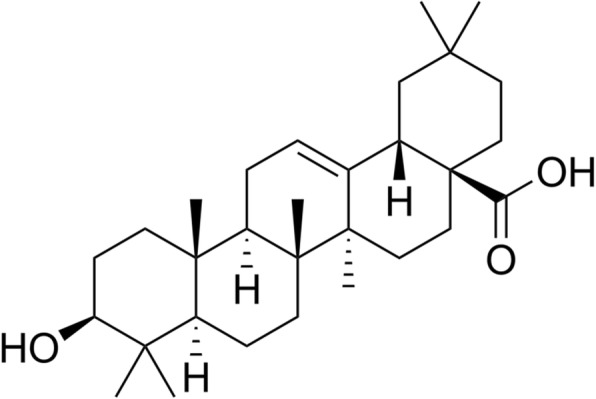
Chemical structure of Oleanolic acid

Oleanolic acid (OA) (Fig. 1) was purchased from Tauto Biotech (Shanghai. China) (Purity higher than 98%). OA was dissolved in DMSO for its administration. Recombinant TGF-β1 was purchased from Peprotech (Cat. No. 100-21C, USA). All other chemicals used in this study were either HPLC or analytical grade.

### Cell culture and treatment

NRK-52E cells were purchased from the Institute of Biochemistry and Cell Biology (Shanghai, China) and cultured in DMEM/F12 (Gibco, USA) with 10% fetal bovine serum (FBS) (Gibco, USA) in an atmosphere of 5% CO_2_ at 37 °C. To determine the effects of OA treatment on the EMT, NRK-52E cells were incubated into 6-well plates with 50–60% confluence were starved for 24 h by incubation with DMEM/F12 containing 0.5% FBS and then divided into following groups: (1) normal control group incubated in DMEM/F12 containing 0.1% DMSO (i.e., vehicle); (2) TGF-β1 group stimulated with recombinant TGF-β1 (5 ng/mL); and (3) OA-treated groups stimulated with recombinant TGF-β1(5 ng/mL) and simultaneously treated with different concentrations of OA (2, 4, and 8 μM). After 48 h, cells were harvested and processed for western blot analysis.

### Western blotting

Western blot assays were used to evaluate the expression of the protein levels. Briefly, cells were lysed in lysis buffer (20 mM Tris, 1 mM EDTA, 1% Triton X-100, 1 mM Na3VO4, 20 mg/ml Aprotinin, 20 mg/ml Leupeptin, 1 mM DTT, and 1 mM PMSF) and the crude protein lysate (40 μg) was resolved by 12% SDS-PAGE. After protein was transferred to a polyvinylidene difluoride (PVDF) membrane, the PVDF membrane was blocked with 5% (*w*/*v*) non-fat milk in Tris buffered saline (TBST) for 1 h at 37 °C. The blots were probed with a dilution of primary antibody. Antibodies used were as follows: anti-fibronectin (ab23751, Abcam, Cambridge, UK), anti-E-cadherin (ab133597, Abcam, Cambridge, UK), anti-α-SMA (ab5694, Abcam, Cambridge, UK), anti-Klotho antibody (ab203576), anti-Nrf2 antibody (ab137550), anti-pSmad2/3 (ab63399, Abcam, Cambridge, UK), anti-Smad2/3 (ab63672, Abcam, Cambridge, UK), anti-ILK (ab137912, Abcam, Cambridge, UK), anti-Snail (ab180714, Abcam, Cambridge, UK), and β-actin (Santa Cruz Biotechnology, Inc.). After hybridization, the blots were washed and hybridized with 1:5000 (*v*/v) dilutions of goat anti-rabbit IgG, horseradish peroxidase-conjugated secondary antibody (Santa Cruz Biotechnology, Inc.). The signal was generated by adding enhanced chemiluminescent reagent, with β-actin used as an internal control.

### Statistical analysis

Data are shown as means ± standard deviation (SD). The statistical difference between groups was determined by the paired Student’s t-test. A *P*-value less than 0.05 was considered significant.

## Results

### Effects of OA on TGF-β1-induced EMT in NRK-52E cells

**Fig. 2:**
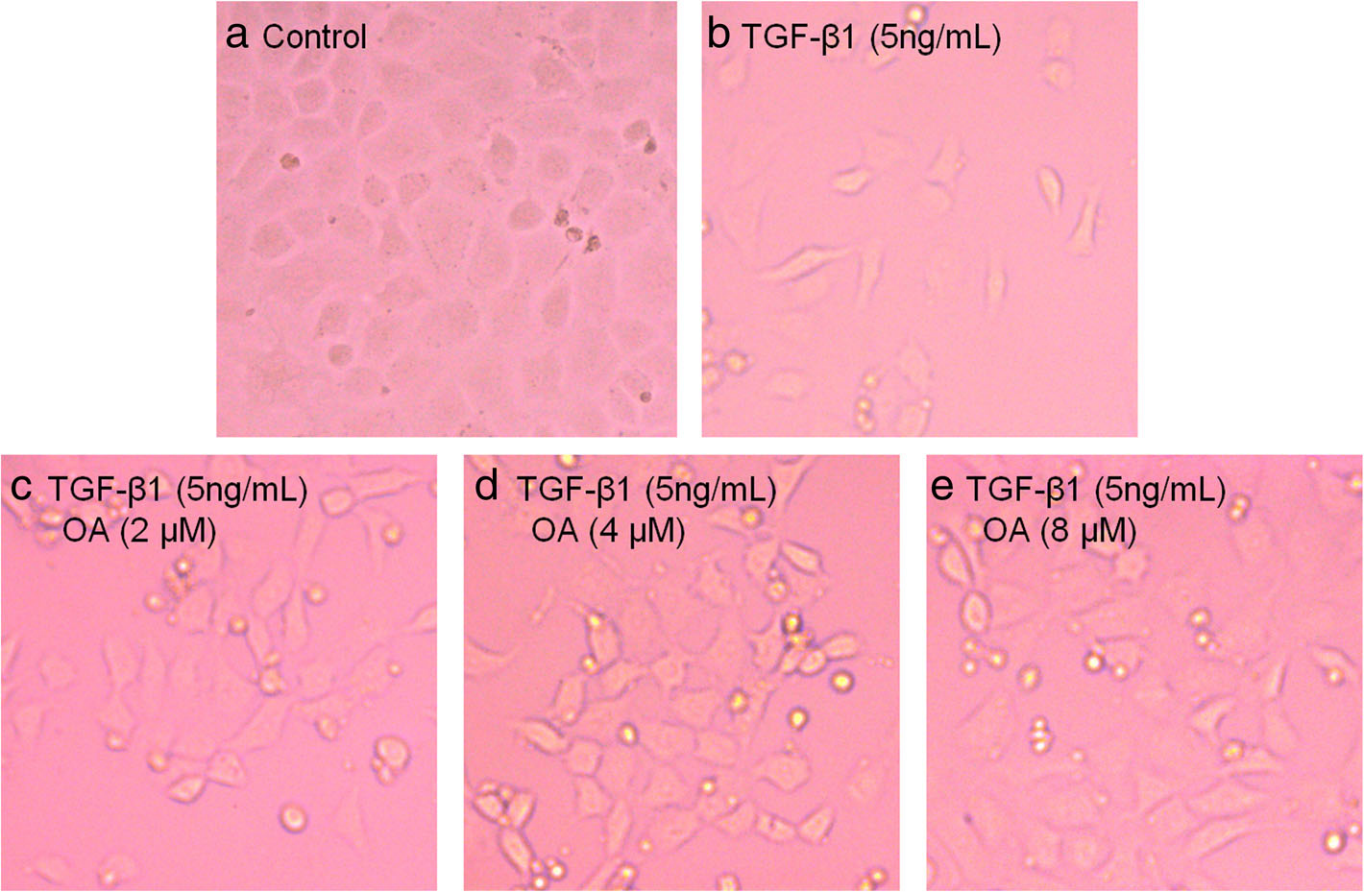
Effects of OA on TGF-β1-induced Morphological changes in NRK-52E cells. The cells were incubated with 5 ng/mL of TGF-β1 for 48 h with different concentrations of OA (0, 2, 4, 8 μM). **a** Untreated NRK-52E cells showed a pebble-like shape is clearly observed. **b** TGF-β1-treated cells showed a more elongated morphological shape. **c**, **d**, **e** showed OA reversed TGF-β1-induced morphological changes (Magnification of 200×)

**Fig. 3:**
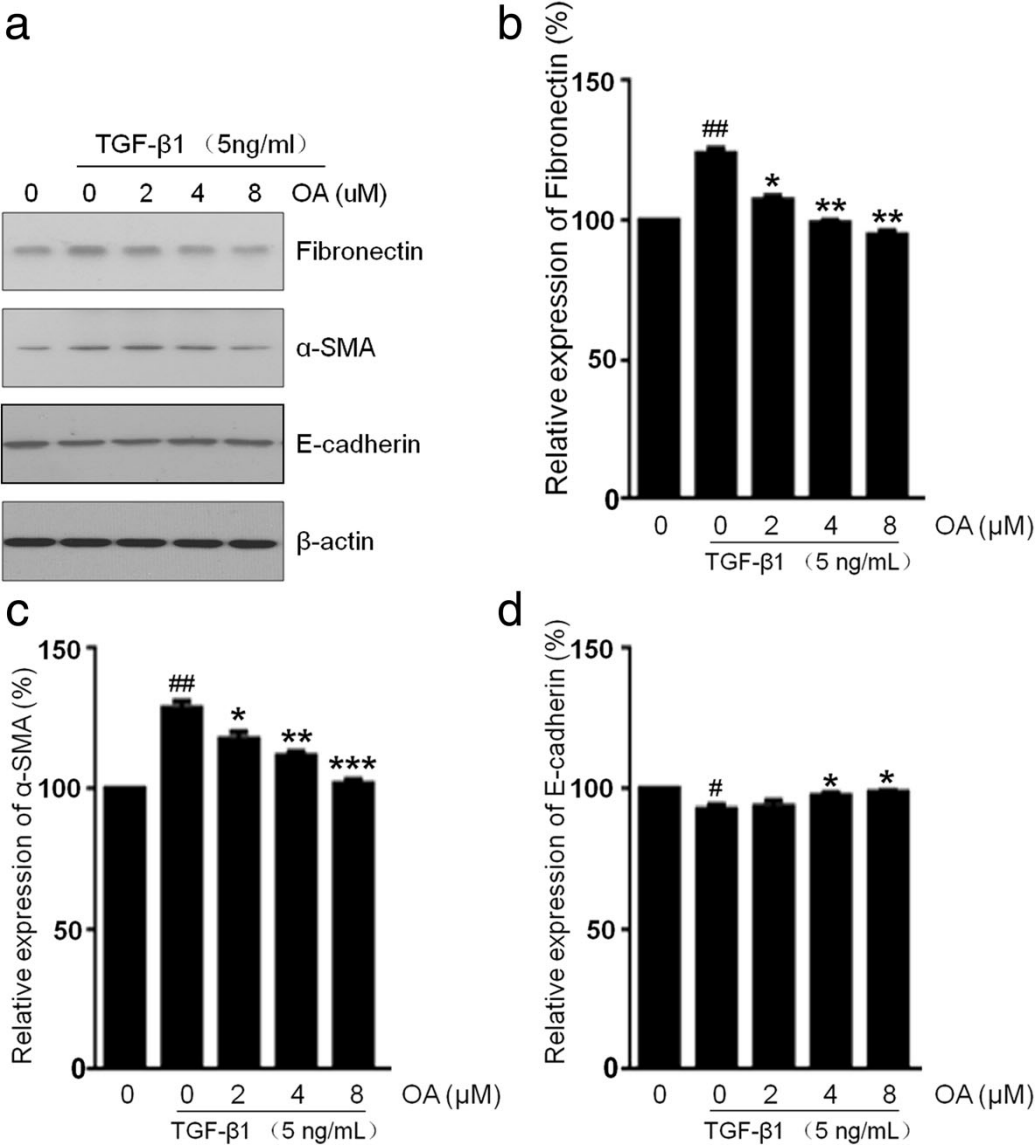
Effects of OA on TGF-β1-induced EMT in NRK-52E cells. The cells were incubated with 5 ng/mL of TGF-β1 for 48 h with different concentrations of OA (0, 2, 4, 8 μM). **a** The expression of Fibronectin, α-SMA and E-cadherin was determined by Western blotting. **b**, **c**, **d** The expression level was quantitatively analyzed with Image J software. The data showing mean ± SD. # *P* < 0.05, ## *P* < 0.01, and ### *P* < 0.005 vs. 0 ng/mL TGF-β1. * P < 0.05, ** P < 0.01, and *** P < 0.005 vs. 0 μM OA in the presence of 5 ng/mL TGF-β1

To determine whether OA suppressed TGF-β1-induced EMT in NRK-52E cells, we studied the cells morphology by Phase-contrast microscopy and the expression level of EMT related proteins by western blotting. The NRK-52E cells were incubated with 5 ng/mL of TGF-β1 for 48 h with different concentrations of OA (0, 2, 4, 8 μM). Phase-contrast microscopy revealed that the cells displayed an elongated, and spindle-like shapes after the incubation with TGF-β1. OA significantly attenuated TGF-β1-induced morphological changes, as shown in Fig. 2. E-cadherin (a classic epithelial cell marker) α-SMA and fibronectin, (the mesenchymal markers), were then analyzed by western blotting. Consistent with the observed morphological changes, the expression of epithelial marker E-cadherin decreased, however the expression of α-SMA, and fibronectin increased after OA treatment in the TGF-β1 treated cells (Fig. 3). These results indicated that OA can suppress TGF-β1-induced EMT in NRK-52E cells.

### Effects of OA on the Nrf2 and klotho expression in NRK-52E cells

**Fig. 4:**
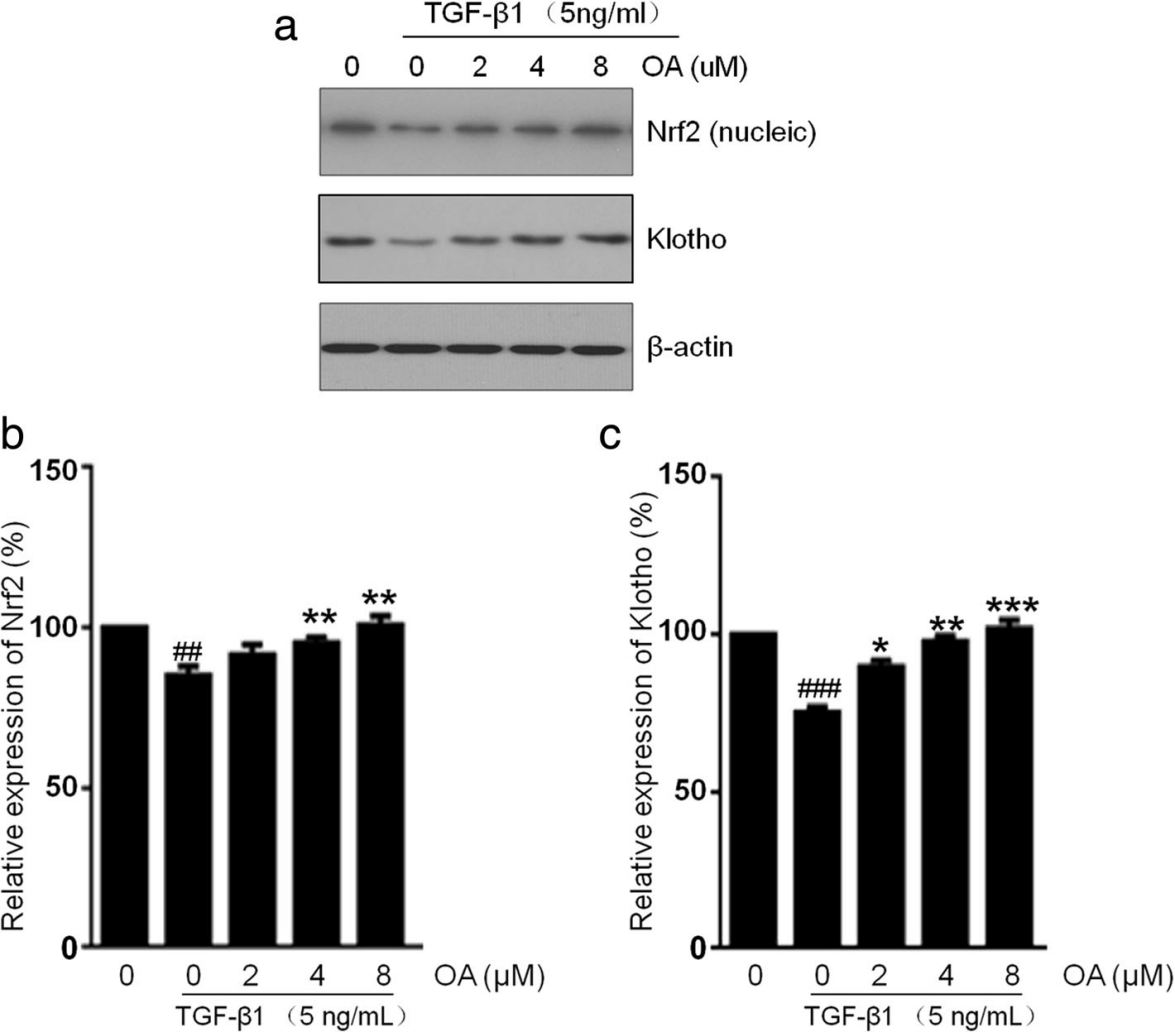
Effects of OA on the Nrf2 and klotho expression in NRK-52E cells. The cells were incubated with 5 ng/mL of TGF-β1 for 48 h with different concentrations of OA (0, 2, 4, 8 μM). **a** The expression level of Nrf2 and klotho was determined by Western blotting. **b**, **c** The expression level was quantitatively analyzed with Image J software. The data showing mean ± SD. # *P* < 0.05, ## *P* < 0.01, and ### P < 0.005 vs. 0 ng/mL TGF-β1. * *P* < 0.05, ** *P* < 0.01, and *** *P* < 0.005 vs. 0 μM OA in the presence of 5 ng/mL TGF-β1

Several studies have shown that Nrf2 activation alleviated renal damage induced by TGF-β1 or toxic in vivo and in vitro [31, 37]. Previous report showed that Nrf2 activation can restore the expression of klotho, an anti-aging protein, suppressing TGF-β1 induced EMT in renal fibrosis, and then attenuate oxidative stress and inflammation in CKD [32].OA is a well-known activator of the transcription factor Nrf2. We investigated whether OA restores the expressions of Nrf2 and klotho downed regulated by TGF-β1. Our result showed that the Nrf2 protein expression decreased after TGF-β1 treatment, however the expression of Nrf2 increases when treated with OA (Fig. 4). The klotho protein expression also decreased after TGF-β1 treatment, but it is restored by the OA treatment. These data demonstrated that OA restores the down-regulation of Nrf2 and klotho mediated by TGF-β1.

### OA attenuates TGF-β1 signaling via Smad2/3 pathway

**Fig. 5:**
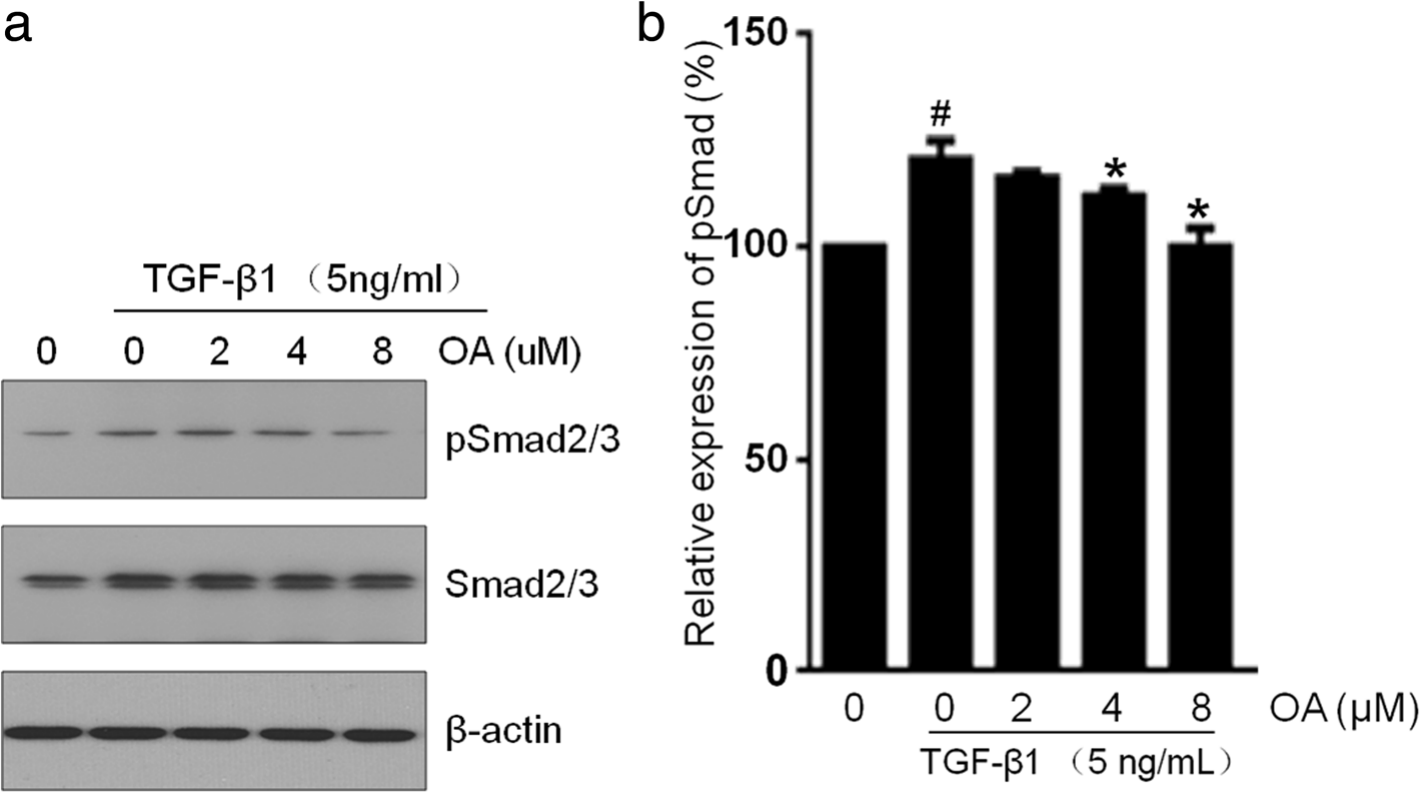
Effects of OA on the expression of pSmad2/3 in NRK-52E cells. The cells were incubated with 5 ng/mL of TGF-β1 for 48 h with different concentrations of OA (0, 2, 4, 8 μM). **a** The expression level of Smad2/3 and pSmad2/3 was determined by Western blotting. **b** The pSmad2/3 was quantitatively analyzed with Image J software. The data showing mean ± SD. # *P* < 0.05 vs. 0 ng/mL TGF-β1. * *P* < 0.05 vs. 0 μM OA in the presence of 5 ng/mL TGF-β1

Activation of smad2/3 by phosphorylation is the central process of the EMT response to TGF-β1 [38]. To address the mechanism by which OA inhibits EMT induced by TGF-β1 in NRK-52E cells, we focused on components downstream of TGF-β1 signaling. Consistent with previous reports, exposure to TGF-β1 resulted in significantly increased Smad2/3 phosphorylation, compared with the vehicle group. Our results also showed that OA treatment significantly decreased the phosphorylation of Smad2/3 in a dose-dependent manner in NRK-52E cells compared with TGF-β1-treated group (Fig. 5). These results showed that the phosphorylation of Smad and the activation of EMT markers induced by TGF-β1 decreased in epithelial cells after OA treatment, suggesting that the TGF-β1/Smads signaling pathways could be responsible for OA-mediated EMT.

### Effects of OA on the ILK and snail expression in NRK-52E cells

**Fig. 6:**
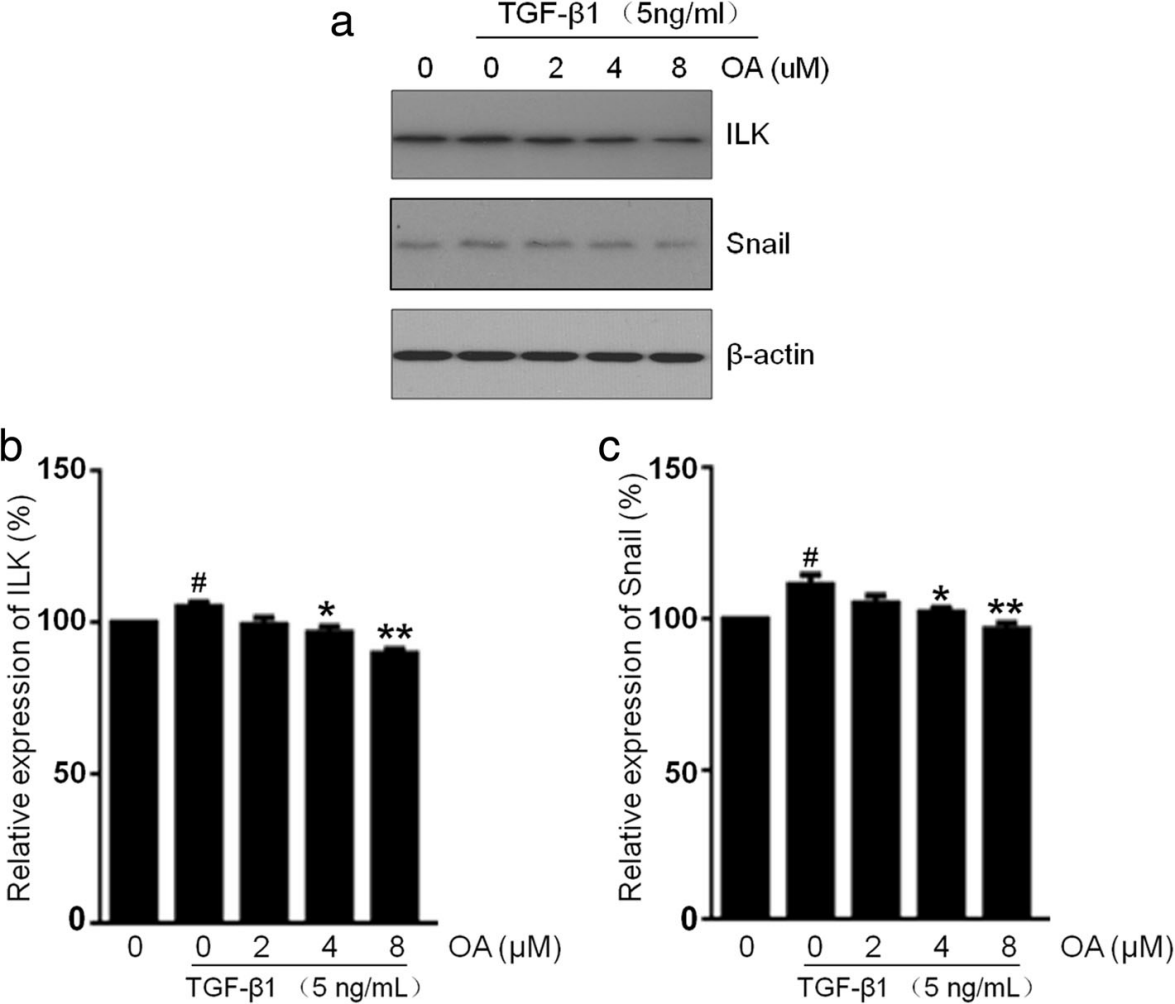
Effects of OA on the ILK and Snail expression in NRK-52E cells. The cells were incubated with 5 ng/mL of TGF-β1 for 48 h with different concentrations of OA (0, 2, 4, 8 μM). **a** The expression level of ILK and Snail was determined by Western blotting. **b**, **c** The expression level was quantitatively analyzed with Image J software. The data showing mean ± SD. # *P* < 0.05 vs. 0 ng/mL TGF-β1. * *P* < 0.05, and ** *P* < 0.01 vs. 0 μM OA in the presence of 5 ng/mL TGF-β1

ILK has been shown to be a key intracellular mediator which controls EMT in tubular epithelial cells by inducing key EMT-regulatory gene Snail expression [18, 19]. To examine whether TGF-β1 promotes ILK and Snail expression and whether OA abolish the EMT process via these process, Western blots were performed. Our results showed that the expressions of ILK and Snail in NRK-52E cells were up-regulated by TGF-β1 but suppressed by OA (Fig. 6).

## Discussion

EMT defines a physiological process that allows polarized epithelial cells converting into motile mesenchymal cells [1–3]. Recent evidences have indicated that EMT may play an important role in the kidney fibrosis. The inhibition of EMT attenuates renal fibrosis induced by TGF-β1, a well-known profibrotic cytokine in the renal fibrosis [10]. Accumulating evidences suggest that OA has beneficially effects on many cellular processes, but its protective activity against EMT remains largely unclear. The model of EMT induced by TGF-β1 in epithelial cells (NRK-52E) has been employed widely in studies of renal fibrosis [39–41]. In this study, we evaluated the effects of OA on EMT of renal proximal tubular epithelial cell line (NRK-52E) induced by TGF-β1, and elucidated its underlying mechanism. In agreement with previous reports on the TGF-β1 response, NRK-52E cells loses their classic cobblestone-like morphology and adopts a mesenchymal spindle-like appearance after treated by TGF-β1. Moreover OA alleviated changes in the expression of these markers induced by TGF-β1, indicating that OA could attenuate TGF-β1-induced EMT in NRK-52E cells.

As we known, OA is a nature potent activator of Nrf2 which has antioxidant activity. Previous reports have shown that Nrf2 can protect fibrosis induced by TGF-β1 via reducing EMT [31]. We investigated the expression level of Nrf2 during the EMT process. The results showed that the expression of Nrf2 in the TGF-β1 treatment group decreased compared to the untreated group, but increased by OA treatment in a dose dependent manner. Klotho is an anti-aging protein predominantly expressed in renal tubular epithelial cells. Evidences showed that the upregulation of Klotho could suppress the EMT process induced by TGF-β1 [35], and Nrf2 activation can restore the expression of klotho and then attenuates oxidative stress and inflammation in CKD [32]. In our study, treatment with OA could restore the expression of klotho in NRK-52E cells which is down-regulated by TGF-β1. The results showed that OA attenuates renal EMT induced by TGF-β1 in NRK-52E cells may be primarily involved the upregulation of Nrf2 and klotho expression.

To further address the mechanism by which OA inhibits EMT in NRK-52E cells induced by TGF-β1, we focused on components downstream of TGF-β1 signaling. TGF-β1/Smads pathway plays a critical role in TGF-β1-induced EMT in epithelial cells. It has been demonstrated that activation of TGF-β1 signaling triggers a dramatic induction of Smad2/3 phosphorylation. Previous reports showed that Nrf2 is involved in the inhibition of smad activation pathway by TGF-β1. Moreover, many reports have shown that klotho suppresses TGF-β1-induced EMT responses in cultured cells, including decreased epithelial marker expression, increased mesenchymal marker expression, and/or increased cell migration by inhibiting TGF-β1/Smads pathway [35]. The results in this study suggests that OA attenuates TGF-β1-induced EMT in NRK-52E cells associated with the modulation of the TGF-β1/Smads pathway. Snail, a key EMT-regulatory gene, downregulates E-cadherin expression and upregulates fibronectin expression, leading to a full EMT phenotype induced primarily by TGF-β1. ILK is well documented as a key intracellular mediator that promotes EMT in tubular epithelial cells by inducing Snail expression [18–21]. Our study demonstrated that ILK and Snail were down-regulated in response to OA during TGF-β1-induced EMT.

The above observation demonstrated that OA attenuates renal EMT processes induced by TGF-β1 in NRK-52E cells. The possible mechanisms involve the upregulation of Nrf2 and klotho expression, suppression of the TGF-β1/Smads pathway and the subsequent inhibition of ILK and Snail expression, and the inhibition of EMT processes. Thus, the anti-fibrotic effect of OA requires further study for future clinical usage.

## Conclusion

In conclusion, we demonstrated that OA, an activator of Nrf2, could dose-dependently attenuate TGF-β1-induced EMT in NRK-52E cells. Therefore, OA may be a potential therapeutic agent which can prevent or attenuate EMT process.

## Abbreviations

bZip: basic leucine-zipper
CKD: Chronic kidney disease
DMEM/F-12: Dulbecco’s Modified Eagle Medium/Nutrient Mixture F-12
DMSO: Dimethyl sulfoxide
DTT: dithiothreitol
EMT: Epithelial-to-mesenchymal transition
FBS: fetal bovine serum
HPLC: High-performance liquid chromatography
Nrf2: NF-E2-related factor 2
OA: Oleanolic acid
PBS: Phosphate buffer saline
PMSF: Phenylmethylsulfonyl fluoride
PVDF: polyvinylidene difluoride
SD: Standard deviation
TBST: Tris buffered saline
TGF-β1: transforming growth factor-β1
α-SMA: α-smooth muscle actin
